# Urinary Incontinence in Midlife According to Weight Changes Across and After Childbearing Years

**DOI:** 10.1007/s00192-023-05713-z

**Published:** 2024-01-12

**Authors:** Katja Taastrøm, Anne Cathrine Kjeldsen, Sarah Hjorth, Ditte Gommesen, Susanne M. Axelsen, Ellen A. Nohr

**Affiliations:** 1 Department of Obstetrics and Gynecology, Aarhus University Hospital, Palle Juul-Jensens Boulevard 99, 8200 Aarhus, Denmark; 2 Research Unit for Gynecology and Obstetrics, Department of Clinical Research, University of Southern Denmark, Odense, Denmark

**Keywords:** Body mass index, Mixed urinary incontinence, Stress urinary incontinence, Urgency urinary incontinence, Weight change

## Abstract

**Introduction and Hypothesis:**

The objective was to investigate how weight change across and after the childbearing years was associated with urinary incontinence (UI) in midlife.

**Methods:**

Data were obtained from 35,645 women responding to the Maternal Follow-up questionnaire in the Danish National Birth Cohort in 2013–2014. Outcome was self-reported UI and its subtypes. Exposures were changes in body mass index (BMI) across and after the childbearing years. Adjusted odds ratios were estimated using logistic regression.

**Results:**

At follow-up, the mean age was 44 years and 32% experienced UI. Compared with stable weight, weight gain across the childbearing years of > 1 to 3, > 3 to 5 or > 5 BMI units increased the odds of any UI by 15%, 27%, and 41% respectively. For mixed UI, the odds increased by 23%, 41%, and 68% in these groups. Weight gain after childbearing showed the same pattern, but with a higher increase in the odds of mixed UI (25%, 60%, and 95% in the respective groups). Women with any weight loss during this period had 9% lower odds of any UI than women with a stable weight.

**Conclusions:**

Weight gain across and after childbearing increased the risk of UI in midlife, especially the subtype mixed UI. Weight loss after childbearing decreased the risk.

**Supplementary information:**

The online version contains supplementary material available at 10.1007/s00192-023-05713-z.

## Introduction

Urinary incontinence (UI), defined as ‘the complaint of involuntary loss of urine’ by the International Continence Society [1], is highly prevalent among women. The condition can have serious implications on physical, psychological and social well-being, and poses economic burdens on individuals and society [2, 3]. Several surveys show self-reported UI in 25–40% of women under the age of 50 years [4, 5], with the most common subtypes being stress urinary incontinence (SUI) and urgency urinary incontinence (UUI). SUI is defined as loss of urine while coughing, sneezing or exercising, and UUI as loss of urine associated with a sudden need to void that is impossible to delay. When both types exist side-by-side, the subtype is called mixed urinary incontinence (MUI) [1, 2]. Although all subtypes affect health-related quality of life, UUI is generally experienced being more bothersome than SUI, as the leakage is unpredictable and often a large quantity [2], whereas MUI is considered to be the most complex condition [6, 7].

An important predisposing factor for UI is high body mass index (BMI) [6]. Studies suggest twice the risk of UI in women with obesity compared with women of normal weight [8]. Substantial weight gain over several years during adulthood was associated with SUI in one study [9], whereas others showed a considerable reduction in UI episodes following weight loss [10, 11]. As aging itself is associated with weight gain [12] and the childbearing years entail weight gain for many women [13, 14], further exploration of the impact of weight change during and after childbearing years on UI is needed. To our knowledge, no studies have investigated the impact of weight changes during childbearing years on the risk of UI. Our aim was to investigate the association between weight change across and after the childbearing years and UI in a large Danish cohort of parous women in midlife.

## Materials and Methods

### Data sources

This prospective cohort study was based on data from the Danish National Birth Cohort (DNBC) [15]. Between 1996 and 2002, a total of 91,381 pregnant women were enrolled in the cohort, recruited at their first antenatal visit to their general practitioner. They were invited to participate in two telephone interviews during pregnancy and another two interviews postpartum. A maternal follow-up took place in 2013–2014 as an online questionnaire completed by 43,639 women (53% of 82,569 eligible women) [16].

### Outcome

All information about UI was obtained from the Maternal Follow-up. A Danish translation of the International Consultation on Incontinence Questionnaire-Female Lower Urinary Tract Symptoms Modules, derived from the validated BFLUTS questionnaire [17], was used to collect information on UUI, SUI, unexplained UI, nocturnal enuresis and frequency of UI (questions presented in Table S1). Each of the five items had five answer categories from 0 to 4, representing the severity of leakage as “not at all, rarely, from time to time, often or every time” and together they formed the outcome Urinary Incontinence Symptoms Subscale (UISS), range 0 to 20.

Stress urinary incontinence, UUI, and MUI were studied as separate outcomes based on the two questions “Does urine leak when you are physically active, exert yourself, cough or sneeze?” and “Does urine leak before you can get to the toilet?” To construct binary variables, the five answer categories were dichotomized. Women who answered “from time to time,” “often,” or “every time” were categorized as being urinary incontinent. Women who answered “not at all” or “rarely” were categorized as not being urinary incontinent. Participants who had symptoms of both SUI and UUI were categorized as having MUI. The variables SUI, UUI, and MUI were mutually exclusive. The outcome any urinary incontinence (any UI), included women categorized as having SUI, or UUI, or MUI.

### Exposures

The study included two exposures: changes in BMI across the childbearing years and changes in BMI after the childbearing years. All BMI measurements were calculated from self-reported information on height from the first interview in the DNBC and weight before the first pregnancy, weight 1 year after the last birth, and weight at follow-up from the Maternal Follow-up. BMI was measured as weight in kilograms/(height in meters)^2^ and categorized according to the definition of the World Health Organization [18] as underweight (BMI < 18.5), normal weight (18.5–24.9), overweight (25–29.9), or obesity ($\ge$ 30).

Changes in BMI across the childbearing years, from before the first pregnancy to 1 year after the last birth, and changes in BMI after the childbearing years, from 1 year after the last birth to follow-up, were calculated as the difference between the two BMI measurements and divided into five categories: weight loss (≤ –1 BMI units), stable weight (> –1 to 1 BMI units) and three categories with weight gain (> 1 to 3, > 3 to 5, and more than 5 units). A weight change of one BMI unit corresponds to approximately 2.8 kg for a woman with a height of 167 cm.

### Covariates

Covariates were chosen a priori based on a literature review. Information about smoking in pregnancy, chronic disease, and socio-occupational status were obtained from the first interview in the DNBC. Chronic disease (yes/no) was based on self-reported information on diabetes, multiple sclerosis, hypertension, and chronic obstructive pulmonary disorder, all predictors of UI [2, 4]. Information on parity, maternal age at first birth, calendar year at first birth, vaginal birth ever, and calendar year at last birth were collected from the Danish Medical Birth Register and linked to the cohort data through the participants’ personal identification numbers. Information on age at follow-up and menopausal status was obtained from the Maternal Follow-up.

### Study Population

**Fig. 1:**
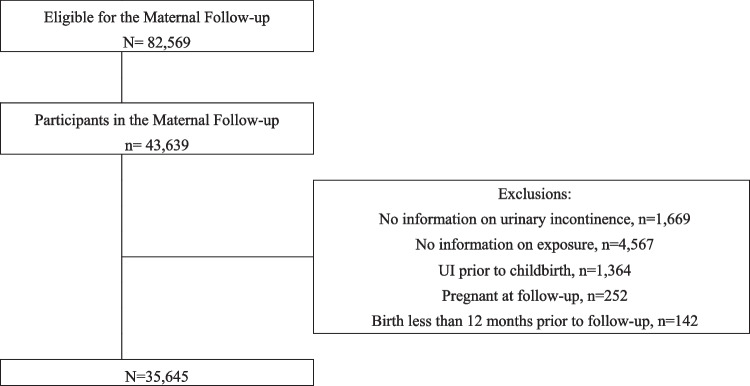
Flowchart of the study population

The 43,639 women who had participated in the Maternal Follow-up were eligible for this study. For all the analyses we excluded 1,669 women who had not answered any questions on UI, and 4,567 women who had not answered questions necessary to construct exposure variables. As pregnancy and the postpartum period itself are considered risk factors for UI, we also excluded women who reported having had UI already prior to childbirth (*n* = 1,364), women who were pregnant at follow-up (*n* = 252), or women with less than 12 months between last birth and follow-up (*n* = 142) [19]. This left us with a study population of 35,645 women (Fig. 1).

### Statistical Analyses

Population characteristics were presented according to BMI changes across the childbearing years as frequencies and percentages.

To provide a graphic presentation of the relationship between BMI changes across and after the childbearing years and UI, we generated restricted cubic splines with six knots for the association between BMI changes as continuous measurements and the UISS score, any UI, SUI, UUI, and MUI. A BMI change of zero was used as the reference in all splines.

We used multiple linear regression to estimate mean differences with 95% confidence intervals on the UISS according to BMI changes. We used multiple logistic regression to estimate odds ratios (OR) with 95% confidence intervals for the association between BMI changes in categories and the outcomes any UI, SUI, UUI, and MUI. Stable weight (> –1 to + 1 BMI units) served as reference group in these analyses.

We controlled for confounders as follows: for changes in BMI across the childbearing years, the main adjusted model included smoking in pregnancy, socio-occupational status, and chronic disease (all categorized as shown in Table 1), maternal age at first birth, and BMI before first pregnancy. In a second adjusted model, we further included parity (categorized as shown in Table 1), calendar year at first birth, calendar year at last birth, and vaginal birth ever. For changes in BMI after the childbearing years, the main adjusted model included the same variables as in the two models above and in addition, BMI 1 year after last birth. As post-menopause could be considered an effect modifier of the association between weight changes and UI, especially UUI and MUI [20], we examined for, but did not find, effect modification by menopausal status.

**Table 1:** Participant characteristics by changes in body mass index (BMI) across the childbearing years

	Changes in BMI across the childbearing years					
	All, *N* = 35,645	≤ −1, *n* = 2,965 (8.3%)	> −1 to 1, *n* = 13,407 (37.6%)	> 1 to 3, *n* = 11,611 (32.6%)	> 3 to 5, *n* = 4,671 (13.1 %)	> 5, *n* = 2,991 (8.4%)
BMI before first pregnancy, *n* (%)						
< 18.5	2,420 (6.8)	54 (1.8)	875 (6.5)	870 (7.5)	373 (8.0)	248 (8.3)
18.5–24.9	27,372 (76.8)	1,897 (64.0)	10,960 (81.7)	9,225 (79.5)	3,390 (72.6)	1,900 (63.5)
25–29.9	4,577 (12.8)	693 (23.4)	1,256 (9.4)	1,232 (10.6)	732 (15.7)	664 (22.2)
≥ 30	1,276 (3.6)	321 (10.8)	316 (2.4)	285 (2.5)	175 (3.7)	179 (6.0)
Age at first birth, *n* (%)						
< 25	6,651 (18.7)	571 (19.3)	1,822 (13.6)	2,018 (17.4)	1,159 (24.8)	1,081 (36.1)
25–29	18,582 (52.1)	1,509 (50.9)	7,116 (53.1)	6,193 (53.3)	2,384 (51.0)	1,380 (46.1)
30–34	8,514 (23.9)	719 (24.2)	3,608 (26.9)	2,804 (24.1)	933 (20.0)	450 (15.0)
≥ 35	1,899 (5.3)	165 (5.6)	862 (6.4)	596 (5.1)	196 (4.2)	80 (2.7)
Smoking in index pregnancy, *n* (%)						
Smoking	4,343 (12.2)	483 (16.3)	1,445 (10.8)	1,338 (11.5)	616 (13.2)	461 (15.4)
Cessation	3,244 (9.1)	257 (8.7)	1,053 (7.9)	1,058 (9.1)	493 (10.6)	383 (12.8)
No smoking	28,059 (78.7)	2,226 (75.1)	10,909 (81.4)	9,215 (79.4)	3,562 (76.3)	2,147 (71.8)
Socio-occupational status, *n* (%)						
High	21,396 (60.0)	1,694 (57.1)	8,684 (64.8)	7,111 (61.2)	2,547 (54.5)	1,360 (45.5)
Middle	11,972 (33.6)	1,070 (36.1)	4,042 (30.1)	3,811 (32.8)	1,755 (37.6)	1,294 (43.3)
Low	2,278 (6.4)	201 (6.8)	681 (5.1)	690 (5.9)	369 (7.9)	337 (11.3)
Chronic disease, *n* (%)^a^	2,996 (8.4)	243 (8.2)	929 (6.9)	917 (7.9)	481 (10.3)	426 (14.2)
Parity, *n* (%)						
P1	3,499 (9.8)	433 (14.6)	1,483 (11.1)	1,018 (8.8)	357 (7.6)	208 (7.0)
P2	18,687 (52.4)	1,539 (51.9)	7,334 (54.7)	6,183 (53.3)	2,324 (49.8)	1,307 (43.7)
P3	11,026 (30.9)	827 (27.9)	3,856 (28.8)	3,703 (31.9)	1,581 (33.8)	1,059 (35.4)
P4 +	2,434 (6.8)	166 (5.6)	735 (5.5)	707 (6.1)	409 (8.8)	417 (13.9)
Vaginal birth, *n* (%)						
Ever	32,515 (91.2)	2,665 (89.9)	12,288 (91.7)	10,643 (91.7)	4,237 (90.7)	2,682 (89.7)
Age at follow-up, *n* (%)						
< 40	6,552 (18.4)	740 (25.0)	2,276 (17.0)	1,959 (16.9)	884 (18.9)	693 (23.2)
40–44	14,894 (41.8)	1,275 (43.0)	5,757 (42.9)	4,883 (42.1)	1,851 (39.6)	1,129 (37.7)
45–49	10,935 (30.7)	754 (25.4)	4,135 (30.8)	3,685 (31.7)	1,465 (31.4)	896 (30.0)
≥ 50	3,264 (9.2)	195 (6.6)	1,239 (9.2)	1,085 (9.3)	471 (10.1)	274 (9.2)
Menopause, *n* (%)	1,539 (4.3)	121 (4.1)	529 (3.9)	502 (4.3)	233 (5.0)	154 (5.1)
Missing values^b^	4,859 (13.6)	323 (10.9)	1,760 (13.1)	1,649 (14.2)	702 (15.0)	425 (14.2)

Owing to the rounding of average numbers across imputed datasets, numbers may vary by one woman

^a^Hypertension, multiple sclerosis, diabetes types 1 and 2

^b^Menopause was not included in the imputation models

#### Supplementary Analyses

Changes in BMI might have different effects on women of normal weight compared with women with overweight or obesity. Therefore, we repeated the analyses restricted to women with BMI < 25 before their first pregnancy in the analyses of changes in BMI across the childbearing years, and women with BMI < 25 1 year after last birth in the analysis of changes in BMI after the childbearing years.

#### Multiple Imputation

When women in the Maternal Follow-up were asked to recall their weight 1 year after their last birth, they could report the precise weight or indicate their weight in intervals of 5 kg up to a weight of 100 kg and in intervals of 10 kg for higher weights. Only 16.5% reported their precise weight. To be able to calculate BMI measurements from weight intervals, we used conditional multiple imputation to obtain an estimate of a specific weight within the reported interval.

Around 8% of the participants had missing data on one or more covariates. Missing data on covariates were imputed using multivariate imputation by chained equations [21] with 50 datasets created. As recommended [22, 23], the imputation model reflected our analysis models by including exposures, outcomes, and covariates. The imputation model for changes in BMI across the childbearing years was also stratified on BMI < 25 before first childbirth, and the model for changes in BMI after the childbearing years was stratified on BMI < 25 1 year after the last birth.

The software STATA17 (StataCorp, College Station, TX, USA) was used to carry out all analyses.

#### Ethical Approval and Consent to Participate

Danish law does not require permission for registry-based studies [24]. The establishment of the DNBC was approved by the Committee on Biomedical Research Ethics (reference no. [KF] 01–471/94). Participants in the DNBC gave written consent to the use of their data for research, both from the cohort and from Danish health and social registries including the Danish Medical Birth Registry and the Danish National Patient Registry. The Danish Data Protection Agency gave permission to use the data (approval no. 2014–41-2848).

## Results

### Participant Characteristics

At follow-up, the 35,645 women in our study population were on average 44 years old and their most recent childbirth had on average occurred 11 years earlier. UI was reported by 32% of the women (21.1% SUI, 2.3% UUI, and 8.4% MUI).

Compared with all the other women, those who had maintained their weight across the childbearing years were more likely to be of high socio-occupational status and to have had a normal BMI before their first pregnancy, and less likely to be smokers, to have a chronic disease, or to be post-menopausal at follow-up. Women who had gained more than 5 BMI units across the childbearing years were more likely to be smokers, to have a chronic disease, to be of low socio-occupational status, to have given birth for the first time at a younger age and to have given birth three or more times, compared with all the other women (Table 1).

### Urinary Incontinence Symptoms Subscale

**Fig. 2:**
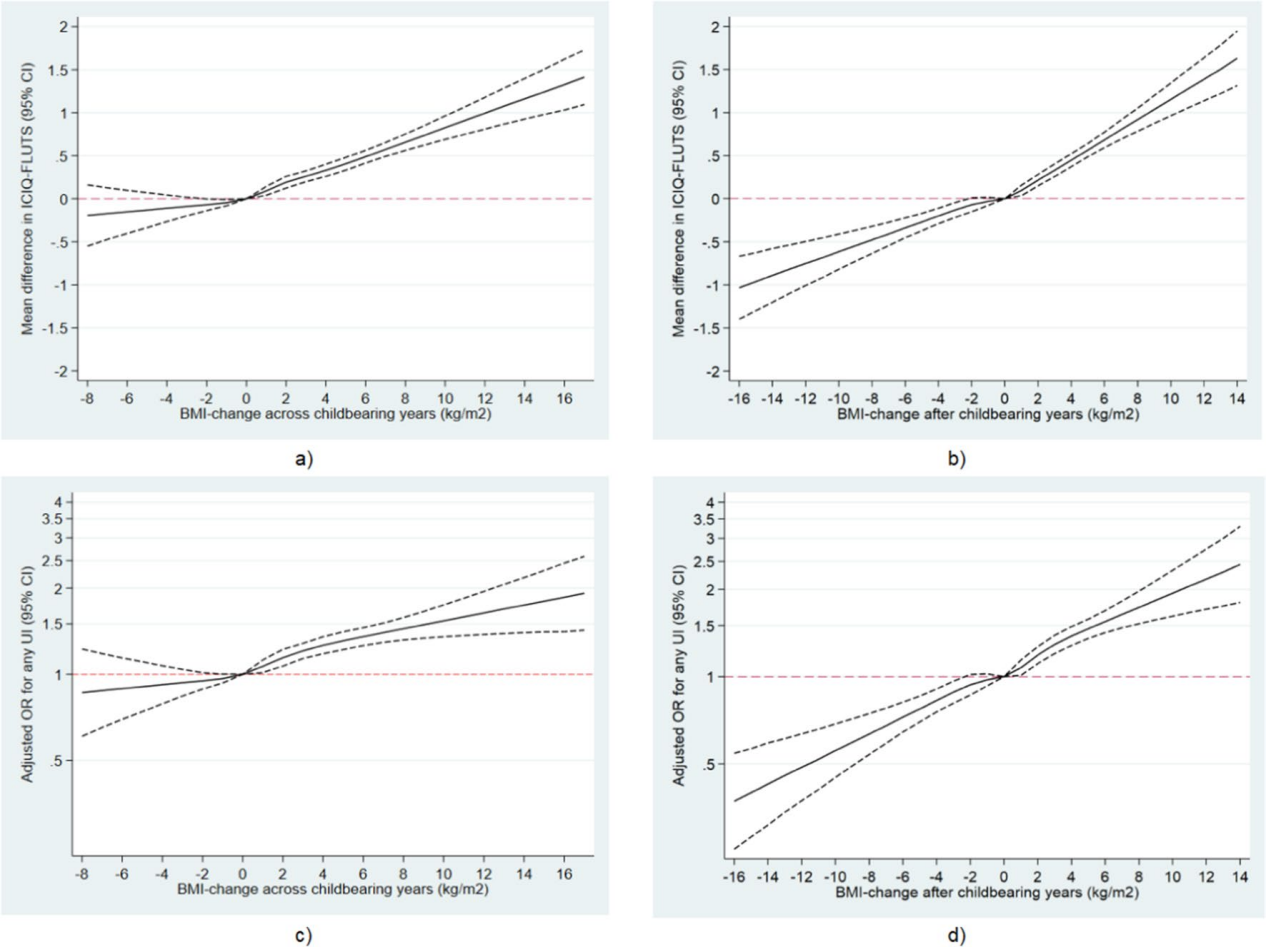
*Upper row*: adjusted mean difference (95% confidence intervals) [*CI*] in symptoms of urinary incontinence (*UI*; International Consultation on Incontinence Questionnaire-Female Lower Urinary Tract Symptoms) according to changes in body mass index (*BMI*) **a** across the childbearing years and **b** after the childbearing years. *Lower row*: adjusted odds ratios (OR) with 95% confidence intervals for any urinary incontinence according to changes in BMI **c** across the childbearing years and **d** after the childbearing years. Analyses across the childbearing years were adjusted for smoking in pregnancy, socio-occupational status, and chronic disease (hypertension, multiple sclerosis, diabetes types 1 and 2) as categorical variables, and maternal age at first birth and BMI before first pregnancy as continuous variables. Analyses after the childbearing years were adjusted for smoking in pregnancy, socio-occupational status, chronic disease (hypertension, multiple sclerosis, diabetes types 1 and 2), and vaginal birth ever as categorical variables and for maternal age at first birth, BMI 1 year after the last birth, parity, calendar year of the first birth and calendar year at the last birth as continuous variables

At follow-up, the mean UISS score was 2.1 (SD 2.2) (distribution shown in Fig. S1). Restricted cubic splines showed linear associations between weight changes across and after the childbearing years and the UISS score (Fig. 2). The association between weight loss and fewer UI symptoms was especially strong after the childbearing years. Using BMI as a continuous variable, we observed an increase in 0.08 points (95% CI) in the UISS for each BMI unit of weight gain across the childbearing years. For changes in BMI after the childbearing years, the corresponding increase was 0.09 points (95% CI).

### Any UI, SUI, UUI, and MUI

#### Changes in BMI across Childbearing Years

**Fig. 3:**
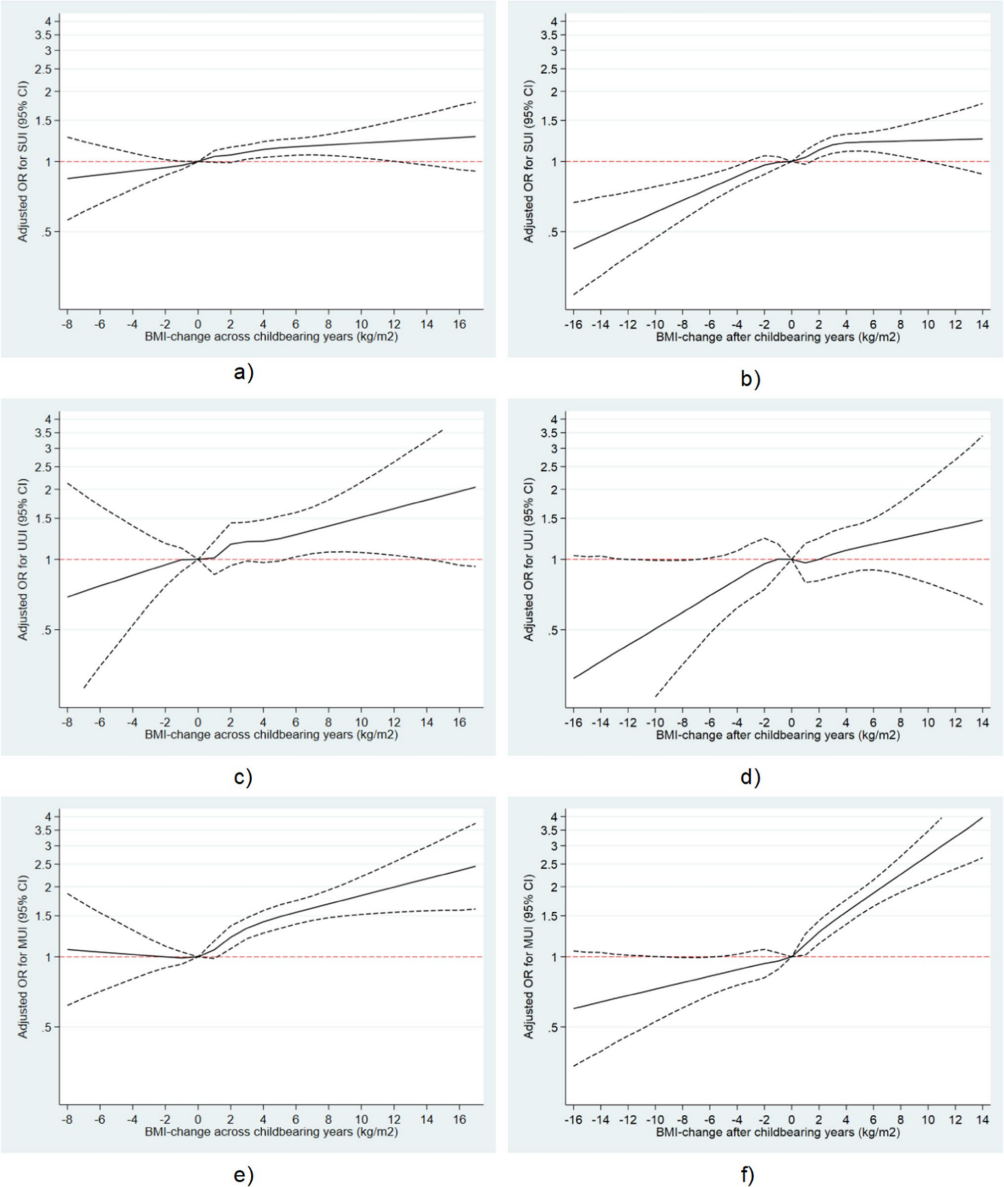
Adjusted odds ratios (*OR*) with 95% confidence intervals for subtypes of urinary incontinence according to changes in body mass index (*BMI*) **a**, **c**, **e** across the childbearing years and **b**, **d**, **f** after the childbearing years. Subtypes were stress urinary incontinence (*SUI*; **a**, **b**), urgency urinary incontinence (*UUI*; **c**, **d**), and mixed urinary incontinence (*MUI*; **e**, **f**). Analyses across the childbearing years were adjusted for smoking in pregnancy, socio-occupational status, and chronic disease (hypertension, multiple sclerosis, diabetes types 1 and 2) as categorical variables, and maternal age at the first birth and BMI before the first pregnancy as continuous variables. Analyses after the childbearing years were adjusted for smoking in pregnancy, socio-occupational status, chronic disease (hypertension, multiple sclerosis, diabetes types 1 and 2), and vaginal birth ever as categorical variables and for maternal age at the first birth, BMI 1 year after the last birth, parity, calendar year of the first birth, and calendar year at the last birth as continuous variables

A strong linear association between weight gain across the childbearing years and increasing OR for any UI was observed in restricted cubic splines, whereas the association between weight loss and lower OR for any UI was weaker and with wide confidence intervals (Fig. 2). The same patterns for weight gain were seen in splines for UUI and MUI, whereas the linear association for SUI was less strong (Fig. 3). For weight loss, patterns for SUI and UUI were similar to those for any UI, but for MUI weight loss did not appear to reduce the OR, although confidence intervals were wide. In the categorical analyses, the prevalence of any UI increased with increasing weight gain from 30% in women with a stable weight to 38% in women who had gained more than 5 BMI units (Table 2). In the main adjusted model, when compared with women who had maintained their weight, the odds of any UI increased by 15%, 27%, and 41% for women who had gained > 1 to 3, > 3 to 5 or more than 5 BMI units respectively. We also observed increased odds with increasing weight gain for all subtypes of UI, lowest for SUI and highest for MUI. Thus, the odds of MUI were increased by 23%, 41%, and 68% for women who had gained > 1 to 3, > 3 to 5, or more than 5 BMI units respectively. Further adjustment for parity and mode of birth had very little impact on these results.

**Table 2:** Odds ratios (*OR*) for urinary incontinence by changes in body mass index (*BMI*) across the childbearing years among women in midlife

	Changes in BMI across the childbearing years				
	≤ −1, *n* = 2,965	> −1 to 1, *,n* = 13,407	> 1 to 3, *n* = 11,611	> 3 to 5, *n* = 4,671	> 5, *n* = 2,991
Any urinary incontinence, *n* = 35,645					
Cases (%)	865 (29.2)	3,949 (29.5)	3,773 (32.5)	1,632 (34.9)	1,122 (37.5)
Crude OR (95% CI)	0.99 (0.89;1.09)	1 (base)	1.15 (1.09;1.22)	1.29 (1.19;1.39)	1.44 (1.32;1.57)
Main adjusted model OR (95% CI)^a^	0.92 (0.84;1.02)	1 (base)	1.15 (1.09;1.23)	1.27 (1.29;1.54)	1.41 (1.29;1.54)
Further adjustments OR (95% CI)^b^	0.92 (0.84;1.02)	1 (base)	1.15 (1.08;1.22)	1.27 (1.17;1.38)	1.42 (1.29;1.55)
Stress urinary incontinence, *n* = 35,645					
Cases (%)	574 (19.4)	2,718 (20.3)	2,516 (21.7)	1,046 (22.4)	681 (22.8)
Crude OR (95% CI)	0.94 (0.84;1.06)	1 (base)	1.09 (1.01;1.17)	1.14 (1.04;1.24)	1.16 (1.05;1.28)
Main adjusted model OR (95% CI)^a^	0.93 (0.83;1.04)	1 (base)	1.09 (1.02;1.17)	1.14 (1.04;1.25)	1.16 (1.05;1.29)
Further adjustments OR (95% CI)^b^	0.92 (0.82;1.04)	1 (base)	1.10 (1.02;1.18)	1.16 (1.06;1.27)	1.21 (1.09;1.34)
Urge urinary incontinence, *n* = 35,645					
Cases (%)	62 (2.1)	280 (2.1)	260 (2.2)	123 (2.6)	88 (2.9)
Crude OR (95% CI)	1.00 (0.73;1.38)	1 (base)	1.07 (0.87;1.32)	1.27 (1.00;1.61)	1.42 (1.10; 1.83)
Main adjusted model OR (95% CI)^a^	0.90 (0.65;1.25)	1 (base)	1.07 (0.87;1.31)	1.24 (0.97;1.57)	1.34 (1.03;1.74)
Further adjustments OR (95% CI)^b^	0.91 (0.65;1.26)	1 (base)	1.05 (0.85;1.29)	1.18 (0.93;1.50)	1.25 (0.96;1.63)
Mixed urinary incontinence, *n* = 35,645					
Cases (%)	228 (7.7)	951 (7.1)	999 (8.6)	462 (9.9)	353 (11.8)
Crude OR (95% CI)	1.09 (0.92;1.29)	1 (base)	1.23 (1.10;1.37)	1.44 (1.26;1.63)	1.75 (1.53;2.01)
Main adjusted model OR (95% CI)^a^	0.98 (0.82;1.16)	1 (base)	1.23 (1.10;1.37)	1.41 (1.24;1.60)	1.68 (1.46;1.94)
Further adjustments OR (95% CI)^b^	0.98 (0.82;1.16)	1 (base)	1.21 (1.09;1.35)	1.36 (1.19;1.54)	1.60 (1.39;1.85)

^a^Adjusted for smoking in pregnancy, socio-occupational status, and chronic disease (hypertension, multiple sclerosis, diabetes types 1 and 2) as categorical variables, and maternal age at the first birth and BMI before the first pregnancy as continuous variables

^b^Adjusted as above and for parity, calendar year at the first birth and calendar year at the last birth as continuous variables, and for vaginal birth ever as the categorical variable

#### Changes in BMI After the Childbearing Years

For weight change from 1 year after last birth to follow-up, restricted cubic splines showed a strong linear association between increasing weight change and OR of any UI across the entire range of weight change. Thus, when compared with women with no weight change, about half the odds of UI was observed in women with weight loss of 10 BMI units and twice the odds in women with weight gain of 10 BMI units (Fig. 2). The same strong pattern was seen for MUI but not for SUI and UUI, where the decrease in OR with weight loss appeared stronger than the increase in OR with weight gain, although confidence intervals were wide (Fig. 3). In categorical analyses, the prevalence of any UI increased from 30% in women with a stable weight to 41% in women with a gain of more than 5 BMI units. In the main adjusted model, when compared with women who had maintained their weight, odds of any UI decreased by 9% in women with weight loss and increased by 17%, 34%, and 59% for women who had gained > 1 to 3, > 3 to 5, or more than 5 BMI units respectively (Table 3). Again, the highest increase in odds with increasing weight gain was seen for MUI, where we observed increased odds of 25%, 60%, and 95% for women who had gained > 1 to 3, > 3 to 5, or more than 5 BMI units respectively.

**Table 3:** Odds ratios (*OR*) for urinary incontinence by changes in body mass index (*BMI*) after the childbearing years among women in midlife

	Changes in BMI after the childbearing years				
	≤ −1, *n* = 7,086	> −1 to 1, *n* = 14,899	> 1 to 3, *n* = 9,076	> 3 to 5, *n* = 3,010	> 5, *n* = 1,574
Any urinary incontinence, *n* = 35,645					
Cases (%)	2,169 (30.6)	4,440 (29.8)	2,991 (33.0)	1,096 (36.4)	644 (40.9)
Crude OR (95% CI)	1.04 (0.97;1.11)	1 (base)	1.16 (1.09;1.23)	1.35 (1.23;1.48)	1.63 (1.46;1.83)
Main adjusted model OR (95% CI)^a^	0.91 (0.85;0.98)	1 (base)	1.17 (1.10;1.24)	1.34 (1.22;1.47)	1.59 (1.42;1.79)
Stress urinary incontinence, *n* = 35,645					
Cases (%)	1,448 (20.4)	3,055 (20.5)	1,989 (21.9)	668 (22.2)	373 (23.7)
Crude OR (95% CI)	1.00 (0.92;1.07)	1 (base)	1.09 (1.01;1.17)	1.11 (0.99;1.23)	1.21 (1.06;1.37)
Main adjusted model OR (95% CI)^a^	0.94 (0.87;1.02)	1 (base)	1.11 (1.03;1.19)	1.14 (1.02;1.28)	1.25 (1.09;1.42)
Urge urinary incontinence, *n* = 35,645					
Cases (%)	169 (2.4)	318 (2.1)	198 (2.2)	81 (2.7)	47 (3.0)
Crude OR (95% CI)	1.12 (0.91;1.38)	1 (base)	1.02 (0.83;1.26)	1.26 (0.96;1.65)	1.42 (1.02;1.97)
Main adjusted model OR (95% CI)^a^	0.95 (0.76;1.19)	1 (base)	0.97 (0.78;1.19)	1.10 (0.83;1.44)	1.16 (0.83;1.63)
Mixed urinary incontinence, *n* = 35,645					
Cases (%)	552 (7.8)	1066 (7.2)	805 (8.9)	347 (11.5)	224 (14.2)
Crude OR (95% CI)	1.09 (0.97;1.23)	1 (base)	1.26 (1.13;1.41)	1.69 (1.46;1.95)	2.15 (1.83;2.53)
Main adjusted model OR (95% CI)^a^	0.91 (0.80;1.03)	1 (base)	1.25 (1.12;1.40)	1.60 (1.38;1.85)	1.95 (1.64;2.31)

^a^Adjusted for smoking in pregnancy, socio-occupational status, chronic disease (hypertension, multiple sclerosis, diabetes types 1 and 2), and vaginal birth ever as categorical variables and for maternal age at the first birth, BMI 1 year after the last birth, parity, calendar year of the first birth, and calendar year at the last birth as continuous variables

### Supplementary Analyses

When restricting the sample to women with a BMI < 25, the estimates for weight change across the childbearing years were very similar to those for all women (Table S2). In the analyses of weight change after the childbearing years, we observed higher odds ratios with weight gain above 5 BMI units for all outcomes except for UUI (Table S3).

## Discussion

### Main Findings

In this large sample of parous women, increasing weight gain across the childbearing years was associated with both an increase in UI in midlife and more severe symptoms of UI. These associations were even stronger for weight gain after the childbearing years, where at the same time a modest reduction in UI was observed in women with weight loss compared with women with a stable weight. Of the UI sub-types studied, MUI showed the strongest association with weight change.

### Strengths and Limitations

A major strength of this study was the large national sample of women with detailed data including a validated questionnaire for symptoms of UI. We also had comprehensive information on potential confounders and weight measurements across the childbearing years until midlife, making it possible to explore BMI changes during several periods in adult life.

A main limitation was the potential misclassification of the weight-change variables. In general, weight tends to be underestimated and height overestimated in self-reported data [13, 25, 26], and all height and weight information in this study was self-reported. Also, in this specific study, the two weight measurements used to generate the weight change across the childbearing years were recalled by the respondents when most of them were in their 40s. We expected that only a few could probably recall their precise weights, but because women often are concerned about their weight, we believed that they would be able to place themselves within 5-kg intervals, an option that was used by 83.5% of the study population. This interval was then imputed into a specific BMI estimate. Even though our exposure variable was a difference between two BMI measures, where we assume both weight measurements to be underreported or overreported to the same degree in the same person, this method would inevitably generate misclassification. However, because we expect this error to be random, or at least not dependent on a woman’s risk of UI, it would bias any association toward the null. Despite these limitations, significant associations between weight changes across the childbearing years and UI were observed. We hope that more accurate data can be collected in future studies that may reveal stronger and more robust associations between weight changes and UI.

Only 53% of eligible participants responded to the Maternal Follow-up and compared with all participants at baseline, they were more likely to be older, of normal weight, and of higher socio-occupational status and less likely to be smokers during pregnancy. Adjusting for factors associated with the status of participation, such as smoking and socio-occupational status, has been shown to limit selection bias [16]. In this study, these factors were accounted for as well. The prevalence of a number of diseases, as well as several exposure-outcome associations, were similar in participants and all those invited to, but not participating in, the Maternal Follow-up, making selection bias concerning UI less likely [16].

We adjusted for a large number of potential confounders but cannot rule out the possibility of residual confounding and the impact of unmeasured confounders. Data to adjust for medication and physical activity at relevant time points were not available. However, apart from BMI measurements, adjustment for other factors had a limited impact on estimates, indicating little confounding of the associations presented.

### Comparison of Findings

In the present study, 32% of the respondents reported UI. This lies within the range of previous studies, suggesting that our results might be generalizable, although the study participants were healthier and of higher socio-occupational status than the background population [27]. Although some did not observe different associations across subtypes [8], others observed a stronger association between BMI and MUI [4, 6, 11, 25, 28], in line with our results for weight gain. The etiology of MUI is not fully understood, but previous findings show that MUI develops either anew or advancing from SUI or UUI. Elderly women and women with obesity are at high risks of both etiological variants [6].

A cohort study with 1,201 participants suggested that women with obesity at age 20 or 26 were at a higher risk of developing severe UI than women who developed obesity at a later age [9]. Our findings do not support this, as we observed slightly higher odds of UI, and especially MUI, in women who were initially of normal weight and gained 5 or more BMI points after the childbearing years compared with the whole population.

Two randomized controlled trials indicated that a 5% weight reduction in women with obesity could noticeably reduce the frequency and severity of UI and improve quality of life [10]. Our data point in the same direction, as weight loss after the childbearing years was associated with a significantly reduced risk of UI.

### Implications

Considerable efforts should be devoted to preventing weight gain in women across and after the childbearing years to reduce the risk of UI in midlife. Health care practitioners should counsel women to reduce postpartum weight retention and maintain a stable weight to prevent the development of UI later in life. Women with symptoms of UI should be informed that even a modest weight loss may reduce symptoms.

## Conclusion

Weight gain, both related to childbearing and after the childbearing years, increased the risk of UI, even in women of normal weight, and was especially associated with MUI, which is considered the most severe subtype of UI. Weight loss after the childbearing years reduced the risk of UI.

### Supplementary information

Below is the link to the electronic supplementary material.

Supplementary file1 (DOCX 62 KB)

## Data Availability

Data may be obtained from the Danish National Birth Cohort on application: https://www.dnbc.dk/access-to-dnbc-data.
