# Supplementary material for: Urinary Incontinence in Midlife According to Weight Changes Across and After Childbearing Years
Source: Int Urogynecol J. 2024 Jan 12;35(3):579–88. doi: 10.1007/s00192-023-05713-z (PMC11024002; doi:10.1007/s00192-023-05713-z)

Supplementary material to

Urinary incontinence in midlife according to weight changes across and after childbearing years - a follow-up study of women in the Danish National Birth Cohort

Katja Taastrøm^1,2*^, Anne Cathrine Kjeldsen^1,2^, Sarah Hjorth^2^, Ditte Gommesen^2^, Susanne M. Axelsen^1^, Ellen A. Nohr^2^

^1^ Department of Obstetrics and Gynecology, Aarhus University Hospital, Aarhus, Denmark

^2^ Research unit for Gynecology and Obstetrics, Department of Clinical Research, University of Southern Denmark, Odense, Denmark

^*^Correspondence: Katja Taastrøm, Palle Juul-Jensens Boulevard 99, 8200 Aarhus, Denmark, tel. 0045 21272774, mail: katalb@rm.dk

Contents:

Table S1: Items and answer categories for urinary incontinence.

Table S2: Odds ratios (OR) for urinary incontinence by changes in body mass index (BMI) across childbearing years among women in midlife, restricted to women with BMI before 1^st^ pregnancy <25

Table S3: Odds ratios for urinary incontinence by changes in body mass index (BMI) after childbearing years among women in midlife, restricted to women with BMI before 1year after last birth<25

Figure S1: Distribution of Urinary Incontinence Symptoms Subscale score

Table S1: Items and answer categories for urinary incontinence.

Based on International Consultation on Incontinence Questionnaire –

Female Lower Urinary Tract Symptoms

Urinary incontinence symptoms subscale (0-20)

|  | Never = 0 | Rarely = 1 | From time to time = 2 | Often = 3 | Every time = 4 |
| --- | --- | --- | --- | --- | --- |
| **1. Urge urinary incontinence/ UUI**  Does urine leak before you can get to the toilet? |  |  |  |  |  |
| **2. Stress urinary incontinence/ SUI**  Does urine leak when you are physically active, exert yourself, cough, or sneeze? |  |  |  |  |  |
| **3. Unexplained urinary incontinence**  Do you ever leak urine for no obvious reason and without feeling that you want to go? |  |  |  |  |  |
| **4. Nocturnal enuresis**  Do you leak urine when you are asleep? |  |  |  |  |  |
|  | Never = 0 | Once or less per week = 1 | Two or three times per week = 2 | 0nce per day =4 | Several times per day = 4 |
| **5. Frequency of urinary incontinence**  How often do you leak urine? |  |  |  |  |  |

**Binary outcomes**

Recoding of **Stress urinary incontinence** (SUI) and **Urge urinary incontinence** (UUI)

into binary outcomes:

Not at all/ rarely = No

From time to time/ Often/ Every time = Yes

**Mixed urinary incontinence (MUI)** comprises **both** UUI **and** SUI.

**Any urinary incontinence** comprises **either** SUI **or** UUI **or** MUI.

| Table S2: Odds ratios (OR) for urinary incontinence by changes in body mass index (BMI) across childbearing years among women in midlife, restricted to women with BMI before 1^st^ pregnancy <25 | | | | | | | |
| --- | --- | --- | --- | --- | --- | --- | --- |
|  |  |  |  |  |  |  |  |
|  |  |  | Changes in BMI across childbearing years | | | | |
|  |  |  | ≤-1 | >-1 to 1 | >1 to 3 | >3 to 5 | >5 |
|  |  |  | 1,946 | 11,829 | 10,085 | 3,761 | 2,144 |
| Any Urinary Incontinence, n=29,765 | | | | | | | |
| Cases (%) | | | 525 (27.0) | 3,408 (28.8) | 3,204 (31.8) | 1,295 (34.4) | 798 (37.2) |
|  |  |  |  |  |  |  |  |
| Crude OR (95% CI) | | | 0.91 (0.81;1.03) | 1 (base) | 1.15 (1.08;1.23) | 1.30 (1.19;1.41) | 1.46 (1.32;1.62) |
| Main adjusted Model OR (95% CI)* | | | 0.90 (0.79;1.01) | 1 (base) | 1.15 (1.08;1.23) | 1.31 (1.20;1.43) | 1.49 (1.34;1.65) |
| Further adjustments OR (95% CI)** | | | 0.90 (0.80;1.01) | 1 (base) | 1.15 (1.08;1.23) | 1.31 (1.20;1.42) | 1.48 (1.33;1.65) |
|  |  |  |  |  |  |  |  |
| Stress Urinary Incontinence, n=29,765 | | | | | | | |
| Cases (%) | | | 358 (18.4) | 2,371 (20.0) | 2,169 (21.5) | 843 (22.4) | 506 (23.6) |
|  |  |  |  |  |  |  |  |
| Crude OR (95% CI) | | | 0.90 (0.78;1.04) | 1 (base) | 1.09 (1.02;1.18) | 1.15 (1.04;1.27) | 1.23 (1.10;1.38) |
| Main adjusted Model OR (95% CI)* | | | 0.89 (0.77;1.02) | 1 (base) | 1.10 (1.02;1.18) | 1.16 (1.05;1.29) | 1.25 (1.11;1.41) |
| Further adjustments OR (95% CI)** | | | 0.88 (0.77;1.02) | 1 (base) | 1.11 (1.03;1.19) | 1.19 (1.07;1.32) | 1.30 (1.15;1.46) |
|  |  |  |  |  |  |  |  |
| Urge Urinary Incontinence, n=29,765 | | | | | | | |
| Cases (%) | | | 39 (2.0) | 243 (2.1) | 215 (2.1) | 97 (2.6) | 56 (2.6) |
|  |  |  |  |  |  |  |  |
| Crude OR (95% CI) | | | 0.98 (0.66;1.46) | 1 (base) | 1.04 (0.83;1.30) | 1.27 (0.98;1.64) | 1.28 (0.94;1.74) |
| Main adjusted Model OR (95% CI)* | | | 0.94 (0.63;1.41) | 1 (base) | 1.04 (0.84;1.30) | 1.27 (0.98;1.66) | 1.29 (0.94;1.77) |
| Further adjustments OR (95% CI)** | | | 0.96 (0.64;1.44) | 1 (base) | 1.02 (0.82;1.27) | 1.20 (0.92;1.56) | 1.17 (0.85;1.62) |
|  |  |  |  |  |  |  |  |
| Mixed Urinary Incontinence, n=29,765 | | | | | | | |
| Cases (%) | | | 128 (6.6) | 794 (6.7) | 819 (8.1) | 354 (9.4) | 236 (11.0) |
|  |  |  |  |  |  |  |  |
| Crude OR (95% CI) | | | 0.97 (0.79;1.21) | 1 (base) | 1.23 (1.09;1.38) | 1.44 (1.25;1.66) | 1.72 (1.46;1.02) |
| Main adjusted Model OR (95% CI)* | | | 0.96 (0.77;1.20) | 1 (base) | 1.23 (1.09;1.39) | 1.45 (1.26;1.67) | 1.73 (1.46;2.05) |
| Further adjustments OR (95% CI)** | | | 0.97 (0.78;1.21) | 1 (base) | 1.21 (1.08;1.37) | 1.39 (1.21;1.61) | 1.62 (1.37;1.92) |
|  |  |  |  |  |  |  |  |
| * Adjusted for smoking in pregnancy, socio-occupational status and chronic disease (hypertension, multiple sclerosis, diabetes type 1 & 2) as categorical variables, and maternal age at 1st birth and BMI before 1st pregnancy as continuous variables. | | | | | | | |
| ** Adjusted as above and for parity, calendar year at first birth and calendar year at last birth as continuous variables and for vaginal birth ever as categorical variable. | | | | | | | |
| Note: Due to rounding of average numbers across imputed datasets, numbers may vary by one woman. | | | | | | | |

| Table S3: Odds ratios for urinary incontinence by changes in body mass index (BMI) after childbearing years among women in midlife, restricted to women with BMI before 1year after last birth<25 | | | | | | | |
| --- | --- | --- | --- | --- | --- | --- | --- |
|  |  |  |  |  |  |  |  |
|  |  |  | Changes in BMI after childbearing years | | | | |
|  |  |  | ≤-1 | >-1 to 1 | >1 to 3 | >3 to 5 | >5 |
|  |  |  | 2,991 | 11,856 | 6,794 | 1,897 | 842 |
| Any Urinary Incontinence, n=24,380 | | | | | | | |
| Cases (%) | | | 828 (27.7) | 3,368 (28.4) | 2,124 (31.3) | 648 (34.2) | 338 (40.1) |
|  |  |  |  |  |  |  |  |
| Crude OR (95% CI) | | | 0.96 (0.87;1.07) | 1 (base) | 1.15 (1.06;1.23) | 1.31 (1.17;1.47) | 1.69 (1.45;1.97) |
| Main adjusted Model OR (95% CI)* | | | 0.91 (0.82;1.01) | 1 (base) | 1.18 (1.09;1.27) | 1.38 (1.22;1.56) | 1.83 (1.65;2.14) |
|  |  |  |  |  |  |  |  |
| Stress Urinary Incontinence, n= 24,380 | | | | | | | |
| Cases (%) | | | 597 (20.0) | 2,341 (19.7) | 1,447 (21.3) | 400 (21.1) | 205 (24.3) |
|  |  |  |  |  |  |  |  |
| Crude OR (95% CI) | | | 1.01 (0.91;1.13) | 1 (base) | 1.10 (1.01;1.20) | 1.08 (0.94;1.24) | 1.31 (1.10;1.55) |
| Main adjusted Model OR (95% CI)* | | | 0.96 (0.86;1.08) | 1 (base) | 1.14 (1.05;1.24) | 1.17 (1.01;1.34) | 1.44 (1.21;1.72) |
|  |  |  |  |  |  |  |  |
| Urge Urinary Incontinence, n=24,380 | | | | | | | |
| Cases (%) | | | 59 (2.0) | 249 (2.1) | 126 (1.9) | 51 (2.7) | 22 (2.6) |
|  |  |  |  |  |  |  |  |
| Crude OR (95% CI) | | | 0.94 (0.67;1.31) | 1 (base) | 0.88 (0.69;1.13) | 1.28 (0.92;1.79) | 1.24 (0.78;1.97) |
| Main adjusted Model OR (95% CI)* | | | 0.91 (0.65;1.28) | 1 (base) | 0.84 (0.65;1.07) | 1.13 (0.80;1.59) | 1.06 (0.66;1.70) |
|  |  |  |  |  |  |  |  |
| Mixed Urinary Incontinence, n=24,380 | | | | | | | |
| Cases (%) | | | 172 (5.8) | 779 (6.6) | 550 (8.1) | 198 (10.4) | 112 (13.3) |
|  |  |  |  |  |  |  |  |
| Crude OR (95% CI) | | | 0.87 (0.71;1.06) | 1 (base) | 1.25 (1.10;1.42) | 1.66 (1.38;1.98) | 2.17 (1.74;2.71) |
| Main adjusted Model OR (95% CI)* | | | 0.82 (0.67;1.01) | 1 (base) | 1.27 (1.12;1.45) | 1.69 (1.40;2.03) | 2.25 (1.78;2.83) |
|  |  |  |  |  |  |  |  |
| * Adjusted for smoking in pregnancy, socio-occupational status and chronic disease (hypertension, multiple sclerosis, diabetes type 1 & 2) and vaginal birth ever as categorical variables and for maternal age at 1st birth, BMI 1 year after last birth, parity, calendar year of 1st birth and calendar year at last birth as continuous variables. | | | | | | | |
| Note: Due to rounding of average numbers across imputed datasets, numbers may vary by one woman. | | | | | | | |

Figure S1: Distribution of Urinary Incontinence Symptoms Subscale score


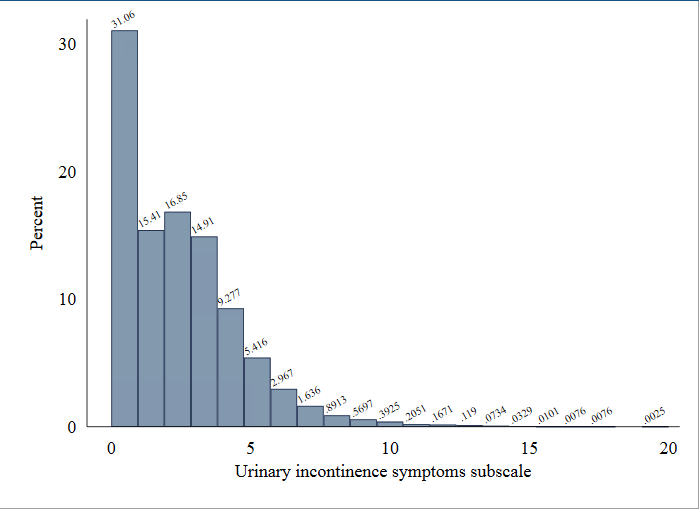

Supplement: Supplementary file 1 — Supplementary file1 (DOCX 62 KB) [file 192_2023_5713_MOESM1_ESM.docx]
